# Do Cryptic Species Exist in *Hoplobatrachus rugulosus*? An Examination Using Four Nuclear Genes, the *Cyt b* Gene and the Complete MT Genome

**DOI:** 10.1371/journal.pone.0124825

**Published:** 2015-04-13

**Authors:** Danna Yu, Jiayong Zhang, Peng Li, Rongquan Zheng, Chen Shao

**Affiliations:** 1 Institute of Ecology, Zhejiang Normal University, Jinhua, 321004, Zhejiang Province, China; 2 College of Chemistry and Life Science, Zhejiang Normal University, Jinhua, 321004, Zhejiang Province, China; 3 Jiangsu Key Laboratory for Biodiversity and Biotechnology, College of Life Sciences, Nanjing Normal University, Nanjing, 210023, Jiangsu Province, China; 4 Institute of Special Aquaculture Source, Zhejiang Normal University, Jinhua, 321004, Zhejiang Province, China; Australian Museum, AUSTRALIA

## Abstract

he Chinese tiger frog *Hoplobatrachus rugulosus* is widely distributed in southern China, Malaysia, Myanmar, Thailand, and Vietnam. It is listed in Appendix II of CITES as the only Class II nationally-protected frog in China. The bred tiger frog known as the Thailand tiger frog, is also identified as *H*. *rugulosus*. Our analysis of the *Cyt b* gene showed high genetic divergence (13.8%) between wild and bred samples of tiger frog. Unexpected genetic divergence of the complete mt genome (14.0%) was also observed between wild and bred samples of tiger frog. Yet, the nuclear genes (*NCX1*, *Rag1*, *Rhod*, *Tyr*) showed little divergence between them. Despite this and their very similar morphology, the features of the mitochondrial genome including genetic divergence of other genes, different three-dimensional structures of ND5 proteins, and gene rearrangements indicate that *H*. *rugulosus* may be a cryptic species complex. Using Bayesian inference, maximum likelihood, and maximum parsimony analyses, *Hoplobatrachus* was resolved as a sister clade to *Euphlyctis*, and *H*. *rugulosus* (BT) as a sister clade to *H*. *rugulosus* (WT). We suggest that we should prevent Thailand tiger frogs (bred type) from escaping into wild environments lest they produce hybrids with Chinese tiger frogs (wild type).

## Introduction

Vertebrate mitochondrial DNA (mtDNA) is a closed circular genome which is approximately 16–20 kb long [1]. This genome typically contains 37 genes (2 rRNAs, 22 tRNAs, and 13 protein-coding genes) and a long non-coding region called the control region or D-loop region [1,2]. The mitochondrial (mt) genome has several valuable characteristics, including small size, fast evolutionary rate, relatively conserved gene content and organization, maternal inheritance, as well as limited recombination [3–6]. Mitochondrial genomes are useful molecular markers in cryptic species identification because of the differences in compositional features, divergence of protein-coding genes, number and size of non-coding regions, and gene arrangement [7–9].

According to the Amphibian Species of the World 5.6, an online reference (19 Mar. 2014) [10], about 6344 species of Order Anura exist worldwide. Among them, 319 species and subspecies can be found in China [11]. The genus *Hoplobatrachus* with five species is important among the Dicroglossini of Dicroglossinae [12–14]. Only one of these species, known as the Chinese tiger frog occurs naturally in wild environments in China [10,11,15]. This frog has been identified as *Hoplobatrachus rugulosus* [13], although some Chinese researchers insist that it should be named *Hoplobatrachus chinensis*, believing this to be a senior synonym of *H*. *rugulosus* [15]. The Chinese tiger frog is listed in Appendix II of CITES as the only Class II nationally-protected frog in China. A bred tiger frog introduced to China from Thailand is called the Thailand tiger frog by Chinese, and has also been identified as *H*. *rugulosus* according to Frost’s taxonomic methods [10]. Thailand tiger frogs are bred in many farmers for local meat consumption. But more and more Thailand tiger frogs have been captured in the field after escaping from farms, which may affect the diversity of local Chinese tiger frogs. Alam et al. [16] found high divergences in *Hoplobatrachus* using *Cyt b*, 12S rRNA, and 16S rRNA genes. They suggested that *H*. *chinensis* (= *H*. *rugulosus*) may be subdivided into more than one species. Pansook et al. [17] found two distinct clades in *H*. *rugulosus* from Thailand using *Cyt b* gene, and also suggested that two distinct species may be present in *H*. *rugulosus*. In this study, we also found a high divergence between Chinese tiger frog and Thailand tiger frog using *Cyt b* gene. A cryptic species complex is a group of organisms that are typically very closely related yet their precise classification and relationships cannot be easily determined, although some can be separated by DNA sequence analyses [18–20]. Using the complete mt genome, Alam et al. [21] and Yu et al. [22] found duplications of the ND5 gene and the control region in *Hoplobatrachus tigerinus* and *H*. *rugulosus*. The current work aimed to investigate further the differences between Chinese tiger frog and Thailand tiger frog, as well as to determine whether cryptic species are present in *H*. *rugulosus*. Accordingly, we determined the complete mt genome sequences of Thailand tiger frog (bred type (BT)), and *then compared the differences in gene arrangement*, *base compositional features*, *and genetic divergences of mt genes between* Chinese tiger frog (wild type (WT)) and Thailand tiger frog (BT). *The* protein structures of the ND5 genes in *Hoplobatrachus* were also compared. Additionally, *we sequenced* nuclear genes (*NCX1*, *Rag1*, *Rhod*, and *Tyr*) of wild and bred tiger frogs to examine their genetic divergence. *We also* performed molecular phylogenetic analyses to discuss the relationship between Chinese tiger frog and Thailand tiger frog, and all available dicroglossids including *Occidozyga martensii*, *based on the 11 mt protein-coding genes using five Ranidae species as out-groups*. We follow the names proposed by Frost et al. [12] to avoid taxonomic confusion.

## Materials and Methods

### Ethical statement

Although the Chinese tiger frog is a protected species, the Chinese tiger frog samples (wild tiger frogs) we used were donated by the Committee of Forest Administrative Bureau of Jinhua (CFABJ), People's Republic of China in 2000 and 2010. The officers of Forest Administrative Bureau of Jinhua have seized these wild frogs, which died during illegal captivity. The Thailand tiger frog samples (bred tiger frogs) were purchased from various farms from Jinhua, Zhejiang province and sacrificed using ether in our laboratory. The husbandry and breeding procedures of the Thailand tiger frogs in the farms were carried out under the Animal Husbandry Law of the People's Republic of China. The study protocol was reviewed and approved by the Committee of Animal Research Ethics of Zhejiang Normal University.

#### Sample and DNA extraction

All samples were stored at—70°C in the Institute of Ecology, Zhejiang Normal University. Information for all samples is shown in S1 Table. Whole genomic DNA was extracted from frozen tissue sample of thigh muscle of tiger frogs using a standard proteinase K/SDS digest extraction method followed by phenol–chloroform isolation and ethanol precipitation [23]. A sample of the Thailand tiger frog (No. THW1) was used to amplify the complete mt genome; other samples were used to amplify the partial *Cyt b* gene.

### Primer design, PCR amplification, and sequencing

*Cyt b* gene was amplified by normal PCR using primers described by Pansook et al. [17]. *NCX1*, *Rag1*, *Rhod*, and *Tyr* genes were amplified by normal PCR using primers described by Che et al. [24] or Freilich et al. [25,26]. We amplified overlapping fragments that covered the entire mt genome of *H*. *rugulosus* (BT) by normal PCR and long-and-accurate PCR (LA-PCR) methods according to Yu et al. [22]. Seven DNA fragments were amplified using seven pairs of highly conserved primers (12STY J/N, 16STY J/N, C1 J/N, C2 J/N, CB-1 J/N, TYC2-C3 J/N, and WC3-ND4L J/N) [22,27–30]. Based on acquired sequence information, four pairs of normal PCR primers (W12S-16S J/N, WC1-C2 J/N, WC2-C3 J/N, and W16S-C1 J/N) and four pairs of LA-PCR primers (WND3-CB J/N, WCR-CR J/N, WND5-ND5 J/N, and 2WD J/N) were designed using Primer Premier 5.0 (Primer Biosoft International) (fragments 1–15 in Table 1) [27,30,31].

**Table 1:** Primer sequences used in this study.

Fragment	Primer	Forward primer sequences (5′→3′)	Reverse primer sequences (5′→3′)	References
1	12 STY J/N	AAAGGTTTGGTCCTAGCCTT	TACCATGTTACGACTTTCCTCTTCT	[28,30]
2	16 STY J/N	AAAGTGGGCCTAAAAGCAGCCA	CTCCGGTCTGAACTCAGATCACGTAGG	[29]
3	C1 J/N	CAACAYYTHTTYTGATTYTTYGG	GTRWANCCNGWRAANARNGG	[27]
4	C2 J/N	GCAGCHTCHCCNATYATRGARGA	CCRCARATYTCWGARCAYTGNCCR	[27]
5	CB-1 J/N	TAYGTYCTNCCNTGRGGNCARATRTC	ARNACNCCNCCNARTTTRTTNGGRAT	[27]
6	TYC2-C3 J/N	ARATTTGYGGRGCAAACCACA	GACTGCWGTATTAAGGAGGGG	[22]
7	WC3-ND4L J/N	CTATATATCAATGATGGCG	CCRTGTGAKCGRGCWGTRGCAA	[22]
8	W12S-16S J/N	TTTTACGCCCATAACACCTA	TGGCTTACACTTACATTTCG	This study
9	W16S-C1 J/N	GGCTTTACTGTCTCCTTTCTCCAAT	TTTAGGTCGGTCGTGAATATGTGAT	This study
10	WC1-C2 J/N	CGTTGCCCACTTCCACTATGT	GGTAAAGGATGCGGAGGGAG	This study
11	WC2-C3 J/N	CTCCGCATCCTTTACCTTAT	GATTAGCGACCAGTATTTTTGA	This study
12	WND3-CB J/N	CCATCTTTACTCCTCCTACGGC	GGGGCATTATTTGACGGGTT	This study
13	WCR-CR J/N	CACACTAACAAGCCAACAAAAGA	AAAGGGTAAGATAGGAACAAACG	This study
14	WND5-ND5 J/N	ATAGCATTCCACTGGTCTTA	AAGGTTCATCTTAGTATTTTCAG	This study
15	2WD J/N	TTCACTCCTGCCAATCCACTGGTTAC	CTGGGTTTCCCTATCGTGTGCTTTT	This study

Notes: Y = C/T, R = A/G, M = A/C, W = A/T, K = G/T, S = G/C, and H = A/T/C.

All PCRs were performed using a MyCycler Thermal Cycler (Bio-Rad, Hercules, CA, USA). TaKaRa Ex-Taq and LA-Taq Kits (Takara Biomedical, Dalian, China) were used for normal PCR and LA-PCR reactions respectively. The normal PCR was carried out in a 50 μl reaction mixture containing 5 μl of buffer (10× concentration), 4 μl of MgCl_2_ (25 mM), 4 μl of dNTP (2.5 mM), 2 μl of each primer (20 μM), 0.25 μl of Ex-Taq polymerase, 30.75 μl of sterile distilled water, and 2 μl of template DNA. The PCR reactions consisted of an initial denaturation at 95°C for 4 min; 35 cycles of denaturation at 94°C for 40 s plus annealing at 48°C to 60°C for 30 s to 60 s and extension at 72°C for 1 min to 2 min; and a final extension at 72°C for 10 min. The LA-PCR was carried out in a 50 μl reaction mixture containing 5 μl of buffer (10× concentration), 4 μl of MgCl_2_ (25 mM), 8 μl of dNTP (2.5 mM), 2 μl of each primer (20 μM), 0.5 μl of LA-Taq polymerase, 26.5 μl of sterile distilled water, and 2 μl of template DNA. The PCR reactions consisted of an initial denaturation at 94°C for 4 min; 35 cycles of denaturation at 94°C for 30 s plus annealing at 55°C to 65°C for 1 min and extension at 68°C for 3 min to 5 min; and a final extension at 72°C for 10 min. The resultant PCR fragments were electrophoresed on 1% agarose gels, and all target DNAs were purified from excised pieces of gel using an AxyPrep DNA Gel Extraction Kit (Axygen Scientific, Inc. SF, CA, USA) for sequencing. The sequence for each fragment was obtained in an automated DNA sequencer (ABI 3730) from both strands. The long fragments were sequenced using specific primer walking of both strands.

### Sequence assembly and analysis

**Fig 1:**
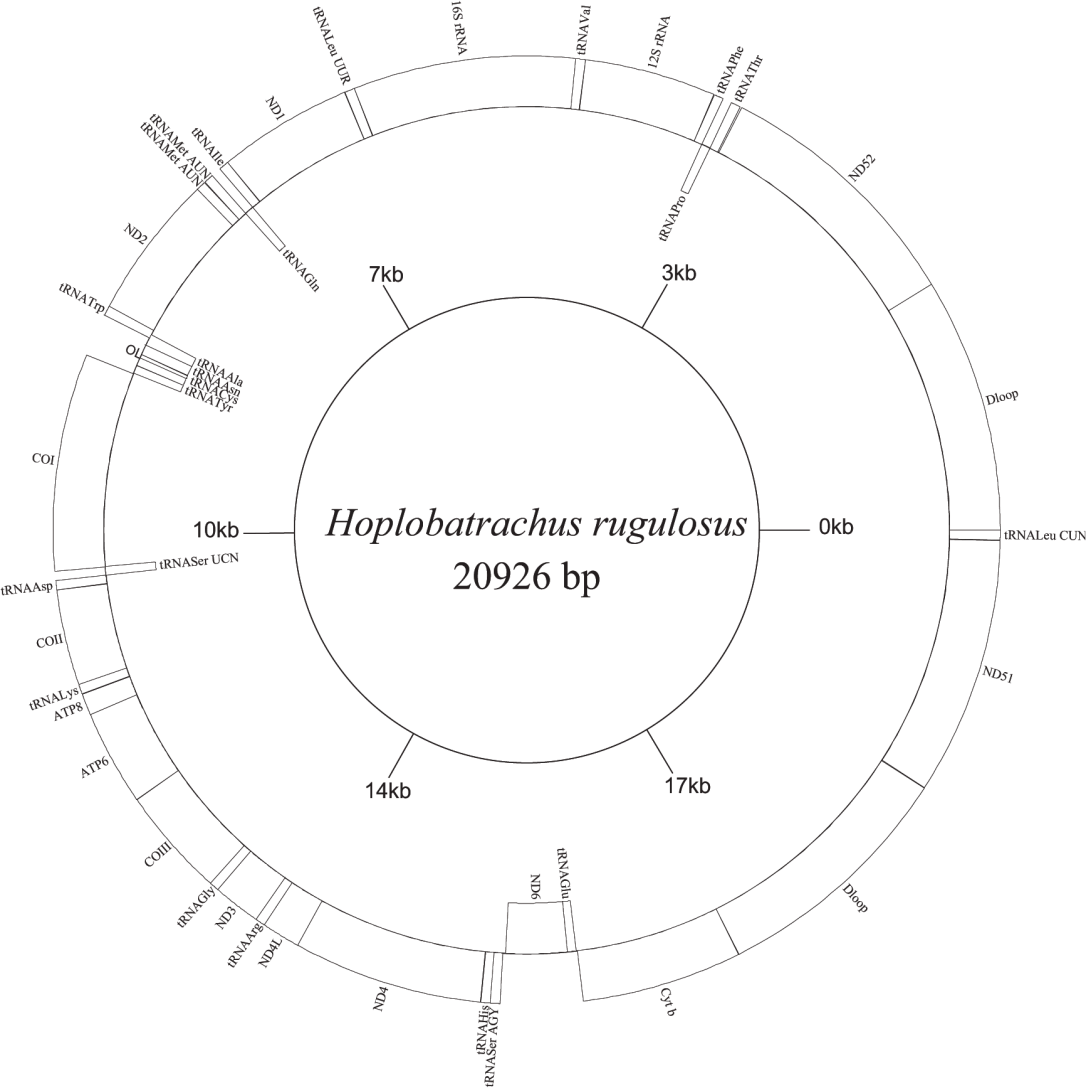
Mitochondrial map of *H*. *rugulosus* bred type. tRNAs are labeled according to the three letter amino acid codes. Gene name inside indicates the direction of transcription from left to right, and gene name outside indicates right to left.

Sequences were checked and assembled using SeqMan (Lasergene version 5.0) [32]. The locations of the 13 protein coding genes and 2 rRNA genes were determined by comparing the homologous sequences of other anurans using Clustal W in Mega 5.0 [25,33]. All tRNA genes, except tRNA^Ser (AGY)^ and tRNA^Cys^ genes, were identified by their cloverleaf secondary structure in tRNA-scan SE 1.21 [34] using the default parameters, and tRNA^Ser (AGY)^ and tRNA^Cys^ genes were determined by comparing the homologous sequences of other anurans. The organization of the *H*. *rugulosus (BT)* mt genome was formed using GenomeVx (Fig 1) (http://wolfe.gen.tcd.ie/GenomeVx/) [35]. The complete mtDNA sequence of *H*. *rugulosus* (BT) reported in this article was deposited in GenBank under accession number JX181763. The haplotypes of *Cyt b*, *NCX1*, *Rag1*, *Rhod*, and *Tyr* genes of 19 frog samples in this study were deposited in GenBank under accession number AB818459-AB818476, KJ637241-KJ637259, KJ637260-KJ637278, KJ637279-KJ63 7297, KJ637298-KJ637316, respectively. The nucleotide sequences of *NCX1*, *Rag1*, *Rhod*, and *Tyr* genes of the Chinese tiger frog and Thailand tiger frog were analyzed by Mega5.0 [25,33].

### Molecular phylogenetic analyses

A total of 55 sequences of the *Cyt b* gene, including 19 from this study, in-group species from Pansook et al. [17] and out-group species (S1 Table), were used to evaluate the divergence between the Chinese tiger frog and the Thailand tiger frog. All 55 sequences yielded 565 bp of *Cyt b* gene fragment, including 231 variable and 197 parsimony-informative sites. Phylogenetic relationships were constructed by neighbor joining (NJ) analysis in Mega 5.0 [33] using Kimura 2-parameter model, gamma distributed, gamma parameter 6, with both transitions and transversions included. Statistical support was evaluated using 1000 bootstrap replicates. Using the data with 20 sequences yielded 1624 bp of *Rag1*, *Rhod*, and *Tyr* genes (excluding *NCX1* gene as no squeence of *Hoplobatrachus* is reported in GenBank), including 36 variable sites. The outgroup was combined *H*. *occipitalis* (*Rag 1* gene HM163613, *Tyr* gene AJ564729) and *H*. *tigerinus* (*Rhod* gene AB489039). Phylogenetic relationships were also constructed by neighbor joining (NJ) analysis in Mega 5.0 [33] with parameter set as above.

To confirm further the phylogenetic relationships of the Chinese tiger frog and the Thailand tiger frog among dicroglossids, 16 available complete mt genomes of anurans based on the addition of two *Hoplobatrachus* mt genomes, including five species of Ranidae ((*Babina adenopleura*, *Pelophylax nigromaculata*, *Pelophylax plancyi*, *Odorrana ishikawae*, and *Odorrana tormotus*) [22,36,37]) as out-groups, were retrieved from GenBank (S2 Table). We constructed the phylogenetic trees based on a concatenated set of 11 mt protein-coding genes excluding ND5 because of the high sequence divergence of its two copies in *H*. *rugulosus* BT and ATP8 because of its low number of informative sites in anurans.

The nucleotide sequences for each of the 11 mt protein-coding genes were aligned using Clustal W in Mega 5.0 [25,33]. To select the conserved regions of the sequences, each alignment was analyzed with Gblocks 0.91b [38] using default settings. We concatenated the alignments of the 11 mt protein-coding genes, and recovered an alignment consisting of 2791 amino acid residues. An alignment of 8373 nucleotides was obtained using the amino acid alignment as the backbone reference. A saturation analysis was performed for subsets with first, second, and third codon positions using DAMBE 4.2.13 [39]. The results showed that the third codon positions were saturated. Consequently, they were excluded from the final nucleotide alignment and an alignment of 5582 nucleotides was obtained. Maximum parsimony (MP) analysis with the nucleotide dataset was performed using PAUP*4.0b10 [40]. A total of 1000 bootstrap replications were generated, each with 10 replications with random taxon order.

Model selection for the nucleotide dataset was performed with Modeltest version 3.7 [41]. The GTR+I+G model was chosen for the likelihood and Bayesian analyses. Maximum likelihood (ML) analysis of the nucleotide dataset was performed using PAUP*4.0b10 [40] with 1000 bootstrap replications. Bayesian inference (BI) analysis of the nucleotide dataset was performed with MrBayes 3.1.2 [42]. Eight chains were run in parallel for 10 000 000 generations, with trees sampled every 1000 generations. The burn-in sizes for both nucleotide datasets were determined by checking convergences of −log likelihood (−lnL) values, and the first 50 000 generations were discarded. Bayesian posterior probabilities were calculated according to the remaining set of trees. All Markov chain Monte Carlo runs were repeated twice to confirm consistent estimation of the posterior parameter distributions.

### Modeling of ND5 Tertiary Structure

A motif scan of ND5 proteins was performed against databases of motifs (http://myhits.isb-sib.ch/cgi-bin/motif_scan) and by using the Simple Modular Architecture Research Tool (http://smart.embl-heidelberg.de/) [43,44]. The Automated Mode program of the Swiss-Model server (http://swissmodel.expasy.org/workspace/index) [45] was used to research the optimization model to enable selection of suitable templates for ND5 proteins. Additional assessments of domain structures were performed on ProSA-Web (https://prosa.services.came.sbg.ac.at/prosa.php) [46,47] and Verify3D Structure Evaluation Server (http://nihserver.mbi.ucla.edu/Verify_3D/) [48–50]. Modeling of the four ND5 protein tertiary structures was performed using Swiss-PdbViewer (http://www.expasy.ch/spdbv/) [50].

## Results

### Genome organization and arrangement

The complete mt genome of *H*. *rugulosus (BT)* was 20 926 bp long and contained 14 protein-coding genes (including the extra copy of the ND5 gene), 2 ribosomal RNAs, 23 transfer RNA genes (including the extra copy of the tRNA^Met^ gene), and 2 non-coding regions (including the extra copy of the control region (D-loop)) (Table 2). In *H*. *rugulosus (BT)*, the distinctive features included a modified cluster of rearranged tRNA genes (TPF tRNA gene cluster), the tandem duplication of tRNA^Met^ genes (Met1 and Met2), the translocation of tRNA^Leu (CUN)^ and ND5 genes, and the two copies of D-loop-ND5 regions. The tRNA^Leu (CUN)^ gene was located between the two D-loop-ND5 regions, rather than in the typical LTPF tRNA cluster (Fig 1 and Table 2). The first D-loop-ND5 region was located between the *Cyt b* and tRNA^Leu (CUN)^ genes, and the second D-loop-ND5 region was located between the tRNA^Leu (CUN)^ and tRNA^Thr^ genes (Fig 1 and Table 2). Most of these genes were coded on the H-strand, except for ND6 and eight tRNA genes, as reported in other anurans (Fig 1).

**Table 2:** Location of features in the mtDNA of *H*. *rugulosus* (BT).

Gene/region	Start position	Stop position	Spacer (+)Overlap (-)	Length (bp)	Start codon	Stop codon	Strand
D-loop (CR2)	1	1815		1815			H
ND5-2	1816	3663	+9	1848	ATG	TAG	H
tRNA^Thr^	3673	3741	-1	69			H
tRNA^Pro^	3741	3809	-1	69			L
tRNA^Phe^	3809	3876		68			H
12S rRNA	3877	4816		940			H
tRNA^Val^	4817	4885		69			H
16S rRNA	4886	6472		1587			H
tRNA^Leu (UUR)^	6473	6545		73			H
ND1	6546	7506		961	ATG	T	H
tRNA^Ile^	7507	7577		71			H
tRNA^Gln^	7578	7648	-1	71			L
tRNA^Met (AUN)^	7648	7719	+3	72			H
tRNA^Met (AUN)^	7723	7791		69			H
ND2	7792	8826	-2	1035	ATG	TAG	H
tRNA^Trp^	8825	8896		72			H
tRNA^Ala^	8897	8965	+2	69			L
tRNA^Asn^	8868	9040		73			L
O_L_	9041	9070	-3	30			L
tRNA^Cys^	9068	9132		65			L
tRNA^Tyr^	9133	9199	+4	67			L
COI	9204	10 754	-9	1551	ATA	AGG	H
tRNA^Ser (UCN)^	10 746	10 816		71			L
tRNA^Asp^	10 817	10 884	+1	68			H
COII	10 886	11 567		682	ATG	T	H
tRNA^Lys^	11 568	11 636	+1	69			H
ATP8	11 638	11 799	-7	162	ATG	TAA	H
ATP6	11 793	12 474		682	ATG	T	H
COIII	12 475	13 260	-1	786	ATG	TAA	H
tRNA^Gly^	13 260	13 328	-3	69			H
ND3	13 326	13 670	-2	345	ATG	TAA	H
tRNA^Arg^	13 669	13 737		69			H
ND4L	13 738	14 019	-7	282	ATG	TAA	H
ND4	14 013	15 369		1357	ATG	T	H
tRNA^His^	15 370	15 438		69			H
tRNA^Ser (AGY)^	15 439	15 506	+23	68			H
ND6	15 530	16 015	+1	486	ATG	AGG	L
tRNA^Glu^	16 017	16 085	+9	69			L
*Cyt b*	16 095	17 240		1146	ATG	TAA	H
D-loop (CR1)	17 241	19 012		1772			H
ND5-1	19 013	20 854	+2	1842	ATG	TAA	H
tRNA^Leu (CUN)^	20 855	20 926		72			H

The overall base composition of the H-strand was as follows: A (26.8%), T (26.0%), G (15.7%), C (31.5%), and total A+T content (52.8%) which was lower than those of other anurans (from 53.1% to 65.3%) (S2 Table). The nucleotide divergence of the complete mt genome was 15.8% between *H*. *rugulosus (WT)* and *H*. *tigerinus*, 18.0% between *H*. *rugulosus (BT)* and *H*. *tigerinus*, and 14.0% between *H*. *rugulosus* (BT) and *H*. *rugulosus* (WT).

### Protein-coding genes

There were six reading frame overlaps in the mt genome of *H*. *rugulosus (BT)*: COI and tRNA^Ser (UCN)^ shared nine nucleotides; ATP8 and ATP6 shared seven nucleotides; ND4L and ND4 shared seven nucleotides; ND2 and tRNA^Trp^ shared two nucleotides; COIII and tRNA^Gly^ shared one nucleotide; and ND3 and tRNA^Arg^ shared two nucleotides. Other overlaps are shown in Table 2. Two ND5 genes (with 84.1% similarity) were found in *H*. *rugulosus (BT) as well as H*. *tigerinus* (two identical ND5 genes) and *H*. *rugulosus* (WT) (two identical ND5 genes). The first ND5 gene (ND5-1) was located between the D-loop and tRNA^Leu (CUN)^, and the second ND5 gene (ND5-2) was located between the D-loop and tRNA^Thr^ (Fig 1 and Table 2). The dataset comparing the two ND5 genes in *H*. *rugulosus* (BT) included 288 variable sites in a total of 1848 aligned nucleotide sites and 131 variable sites in amino acid sequence over a total of 616 alignment amino acid sites. The codon frequency of the two ND5 genes in *H*. *rugulosus* (BT) *is shown in* *Table* 3.

**Table 3:** The RSCU in two different ND5 genes of *H*. *rugulosus* (BT).

	Codon	Count	RSCU	Codon	Count	RSCU	Codon	Count	RSCU	Codon	Count	RSCU
ND5-2	UUU(F)	16	0.64	UCU(S)	11	1.22	UAU(Y)	5	0.71	UGU(C)	3	0.86
ND5-1	UUU(F)	11	0.5	UCU(S)	14	1.47	UAU(Y)	5	0.67	UGU(C)	2	0.57
ND5-2	UUC(F)	34	1.36	UCC(S)	17	1.89	UAC(Y)	9	1.29	UGC(C)	4	1.14
ND5-1	UUC(F)	33	1.5	UCC(S)	20	2.11	UAC(Y)	10	1.33	UGC(C)	5	1.43
ND5-2	UUA(L)	8	0.5	UCA(S)	13	1.44	UAA(*)	0	0	UGA(W)	12	1.5
ND5-1	UUA(L)	11	0.67	UCA(S)	9	0.95	UAA(*)	1	4	UGA(W)	15	1.76
ND5-2	UUG(L)	0	0	UCG(S)	5	0.56	UAG(*)	1	4	UGG(W)	4	0.5
ND5-1	UUG(L)	1	0.06	UCG(S)	6	0.63	UAG(*)	0	0	UGG(W)	2	0.24
ND5-2	CUU(L)	25	1.56	CCU(P)	5	0.65	CAU(H)	3	0.26	CGU(R)	0	0
ND5-1	CUU(L)	21	1.27	CCU(P)	5	0.54	CAU(H)	3	0.29	CGU(R)	1	0.31
ND5-2	CUC(L)	40	2.5	CCC(P)	11	1.42	CAC(H)	20	1.74	CGC(R)	6	1.6
ND5-1	CUC(L)	39	2.36	CCC(P)	15	1.62	CAC(H)	18	1.71	CGC(R)	7	2.15
ND5-2	CUA(L)	14	0.88	CCA(P)	14	1.81	CAA(Q)	9	1.8	CGA(R)	9	2.4
ND5-1	CUA(L)	19	1.15	CCA(P)	17	1.84	CAA(Q)	11	1.57	CGA(R)	5	1.54
ND5-2	CUG(L)	9	0.56	CCG(P)	1	0.13	CAG(Q)	1	0.2	CGG(R)	0	0
ND5-1	CUG(L)	8	0.48	CCG(P)	0	0	CAG(Q)	3	0.43	CGG(R)	0	0
ND5-2	AUU(I)	15	0.73	ACU(T)	9	0.8	AAU(N)	7	0.88	AGU(S)	0	0
ND5-1	AUU(I)	21	0.93	ACU(T)	8	0.74	AAU(N)	5	0.48	AGU(S)	1	0.11
ND5-2	AUC(I)	26	1.27	ACC(T)	23	2.04	AAC(N)	9	1.13	AGC(S)	8	0.89
ND5-1	AUC(I)	24	1.07	ACC(T)	20	1.86	AAC(N)	16	1.52	AGC(S)	7	0.74
ND5-2	AUA(M)	13	1.04	ACA(T)	12	1.07	AAA(K)	13	1.37	AGA(*)	0	0
ND5-1	AUA(M)	13	1.24	ACA(T)	12	1.12	AAA(K)	14	1.33	AGA(*)	0	0
ND5-2	AUG(M)	12	0.96	ACG(T)	1	0.09	AAG(K)	6	0.63	AGG(*)	0	0
ND5-1	AUG(M)	8	0.76	ACG(T)	3	0.28	AAG(K)	7	0.67	AGG(*)	0	0
ND5-2	GUU(V)	10	1.18	GCU(A)	12	0.77	GAU(D)	4	0.5	GGU(G)	1	0.15
ND5-1	GUU(V)	9	1.03	GCU(A)	14	1.08	GAU(D)	5	0.91	GGU(G)	1	0.16
ND5-2	GUC(V)	16	1.88	GCC(A)	29	1.87	GAC(D)	12	1.5	GGC(G)	11	1.69
ND5-1	GUC(V)	16	1.83	GCC(A)	21	1.62	GAC(D)	6	1.09	GGC(G)	10	1.6
ND5-2	GUA(V)	6	0.71	GCA(A)	17	1.1	GAA(E)	10	1.33	GGA(G)	9	1.38
ND5-1	GUA(V)	9	1.03	GCA(A)	16	1.23	GAA(E)	11	1.47	GGA(G)	6	0.96
ND5-2	GUG(V)	2	0.24	GCG(A)	4	0.26	GAG(E)	5	0.67	GGG(G)	5	0.77
ND5-1	GUG(V)	1	0.11	GCG(A)	1	0.08	GAG(E)	4	0.53	GGG(G)	8	1.28

All frequencies are averages over two ND5 genes in *H*. *rugulosus* (BT), and relative synonymous codon usage is given in the column following the codon count column.

Protein-coding genes in *H*. *rugulosus* (BT) begin with ATG as the start codon, except COI with ATA and the ND6 gene, for which *H*. *rugulosus* (WT) used ACA—*H*. *rugulosus* (BT) used ATG. ND5-1, ATP8, COIII, ND3, ND4L, and *Cyt b* genes are terminated with TAA as the stop codon, COI and ND6 end with AGG, ND2 and ND5-2 end with TAG, and the other four protein-coding genes end with an incomplete stop codon (a single stop nucleotide T) (Table 2). In the ND5-2 gene, *H*. *rugulosus* (WT) used TAA as the stop codon, whereas *H*. *rugulosus* (BT) used TAG (Table 2).

Three protein-coding genes surprisingly showed different lengths between *H*. *rugulosus* WT and BT: ND3 differed by 3 bp (one amino acid), ND5-1 differed by 6 bp (two amino acids), and ND5-2 differed by 12 bp (four amino acids).

The divergence of nucleotides and amino acids in protein-coding genes using the uncorrected *p*-distance model between *H*. *rugulosus* (WT) and *H*. *rugulosus* (BT) ranged from 11.2% (COIII) to 24.0% (ND5-2) and from 2.1% (COIII) to 28.3% (ATP8), respectively (Table 4). The divergence (uncorrected *p*-distances) of nucleotides and amino acids in protein-coding genes among *Hoplobatrachus* ranged from 11.2% (COIII) to 28.2% (ND5-2) and from 1% (COI) to 30.5% (ND6), respectively (Table 4). The nucleotide divergence of the two pairs of ND5 genes in *H*. *rugulosus* (WT) and *H*. *rugulosus* (BT) was 18.8% and 24.0%, respectively, and the divergences in amino acid sequence were 14.2% and 26.8%.

**Table 4:** Statistics describing the divergence (*p*-distance) in mitochondrial genes between *H*. *rugulosus* wild type and *H*. *tigerinus*, between *H*. *rugulosus* wild type and bred type, as well as between *H*. *rugulosus* bred type and *H*. *tigerinus*. W: *H*. *rugulosus* wild type; B: *H*. *rugulosus* bred type; T: *H*. *Tigerinus*.

Name of Gene or D-loop	No. of nucleotide differences			Pairwise distances of nucleotide			No. of amino acid differences			Pairwise distances divergence of amino acid		
	W-T	W-B	B-T	W-T	W-B	B-T	W-T	W-B	B-T	W-T	W-B	B-T
D-loop-2	232	225	350	0.156	0.151	0.222						
ND5-2	375	347	423	0.205	0.188	0.232	75	87	107	0.123	0.142	0.176
tRNA^Thr^	8	8	10	0.114	0.114	0.145						
tRNA^Pro^	6	11	12	0.087	0.159	0.174						
tRNA^Phe^	2	4	4	0.029	0.059	0.059						
12S rRNA	61	71	80	0.065	0.076	0.086						
tRNA^Val^	8	10	12	0.116	0.145	0.174						
16S rRNA	128	105	139	0.081	0.066	0.088						
tRNA^Leu (UUR)^	5	0	5	0.068	0	0.068						
ND1	154	142	187	0.161	0.148	0.195	15	23	25	0.047	0.071	0.078
tRNA^Ile^	4	3	3	0.056	0.042	0.042						
tRNA^Gln^	6	10	8	0.085	0.141	0.113						
tRNA^Met (AUN)^	6	6	9	0.083	0.083	0.125						
tRNA^Met (AUN)^	2	1	3	0.029	0.014	0.043						
ND2	208	182	208	0.201	0.176	0.201	38	35	44	0.110	0.102	0.128
tRNA^Trp^	2	4	1	0.029	0.052	0.014						
tRNA^Ala^	6	2	5	0.087	0.029	0.072						
tRNA^Asn^	3	2	3	0.041	0.027	0.041						
O_L_	1	1	2	0.034	0.034	0.069						
tRNA^Cys^	1	7	6	0.015	0.108	0.092						
tRNA^Tyr^	3	1	4	0.045	0.015	0.060						
COI	236	198	248	0.152	0.128	0.160	5	11	14	0.010	0.021	0.027
tRNA^Ser (UCN)^	1	1	2	0.014	0.014	0.029						
tRNA^Asp^	3	9	8	0.044	0.132	0.118						
COII	104	89	120	0.152	0.130	0.176	5	9	10	0.022	0.040	0.044
tRNA^Lys^	2	1	1	0.029	0.014	0.014						
ATP8	40	29	42	0.247	0.179	0.259	13	15	16	0.245	0.283	0.302
ATP6	123	123	138	0.180	0.180	0.202	21	24	21	0.093	0.106	0.093
COIII	116	88	123	0.148	0.112	0.156	3	11	11	0.011	0.042	0.042
tRNA^Gly^	3	6	7	0.043	0.087	0.101						
ND3	66	52	67	0.194	0.152	0.196	9	18	13	0.080	0.160	0.115
tRNA^Arg^	8	2	8	0.116	0.029	0.116						
ND4L	54	44	50	0.191	0.156	0.177	8	5	7	0.086	0.054	0.075
ND4	277	211	292	0.204	0.155	0.215	63	51	76	0.139	0.113	0.168
tRNA^His^	10	10	14	0.145	0.145	0.203						
tRNA^Ser (AGY)^	3	4	1	0.044	0.059	0.015						
ND6	85	56	103	0.175	0.177	0.212	17	16	25	0.105	0.099	0.155
tRNA^Glu^	3	3	4	0.043	0.043	0.058						
*Cyt b*	179	171	209	0.156	0.149	0.182	18	20	23	0.047	0.052	0.060
D-loop-1	232	548	454	0.156	0.362	0.257						
ND5-1	374	440	520	0.205	0.240	0.282	75	163	186	0.123	0.268	0.305
tRNA^Leu (CUN)^	5	5	0	0.069	0.069	0						

### Ribosomal and transfer RNA genes

**Fig 2:**
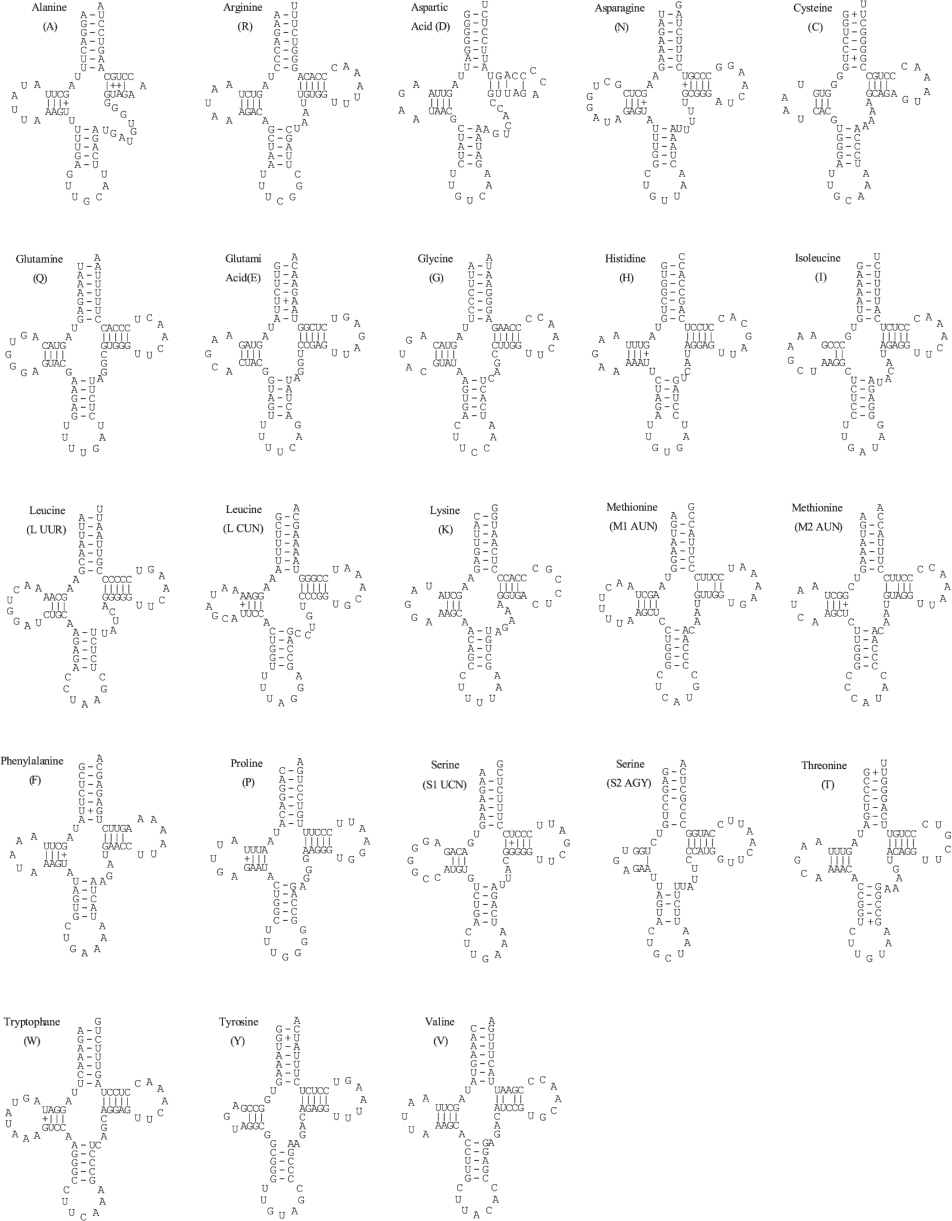
Predicted secondary structures for the 22 tRNA genes of *H*. *rugulosus* bred type. Dashes (–) indicate Watson–Crick base pairing and plus (+) indicate G+U base pairing. Arms of tRNAs (clockwise from the top) are the amino acid acceptor (AA) arm, the TyC (T) arm, the anticodon (AC) arm, and the dihydrouridine (DHU) arm.

In *H*. *rugulosus* (WT), the 12S rRNA gene (940 bp long) was located between tRNA^Phe^ and tRNA^Val^ genes, and the 16S rRNA gene (1587 bp long) was located between tRNA^Val^ and tRNA^Leu (UUR)^ genes. The mt genome of *H*. *rugulosus* (BT) contained 23 tRNA genes (including the extra copy of tRNA^Met^ gene) (Fig 2), that were interspersed in the genome and ranged in size from 65 bp to 73 bp. As reported in anurans, all tRNA genes can be folded into typical cloverleaf secondary structures with the known exception of the tRNA^Ser(AGY)^ gene (Fig 2).

The divergence of 12S rRNA and 16S rRNA genes between *H*. *rugulosus* (WT) and *H*. *rugulosus* (BT) was 7.6% and 6.6%, respectively, using the uncorrected *p*-distance model. Table 4 shows the divergences (uncorrected *p*-distances) of the 23 tRNA genes, 12S rRNA genes, and 16S rRNA genes among *H*. *tigerinus* and *H*. *rugulosus* (WT and BT).

### Noncoding regions

The non-coding regions of *H*. *rugulosus* (BT) included the duplicated control regions (D-loop) and a few intergenic spacers. In *H*. *rugulosus* (BT), the first control region (CR1) was located between the *Cyt b* and ND5 genes, whereas in *H*. *tigerinus* and *H*. *rugulosus* (WT), the second control region (CR2) was located between the tRNA^Leu (CUN)^ and ND5 genes (Fig 1 and Table 2). The two control regions had almost identical nucleotide sequences, except the extra A (or C)AATATGCT_5_ in CR2. The length of control regions was similar to those of other anurans (from 851 bp to 4704 bp). However, the A+T content of the control regions was 57.0% or 56.6% (A, 29.0% or 28.8%; C, 28.7% or 29.0%; G, 14.3% or 14.4%; T, 28.0% or 27.8% in CR1 and CR2, respectively), which was between 54.9% and 73.3% in other anurans (S2 Table). The two control regions contained TASs (5′-ACATTAACTTTCTGT-3′), CSBs (CSB-1, 5′-AGCCCCTATTAATGCTTGATGGACATAG-3′; CSB-2, 5′-GAC CCCCCCCTTACCCCCCCC-3′; CSB-3, 5′-CCTTAGCCCCCCCGAGC-3′), as well as 7 and 12 tandem repeat units of 5′-TCAATATGC-3′ in CR1 and CR2, respectively. Some short non-coding sequences also occurred in *H*. *rugulosus (BT)*, and the longest intergenic space (about 23 bp) was between tRNA^Ser (AGY)^ and ND6 gene (Table 2).

**Fig 3:**
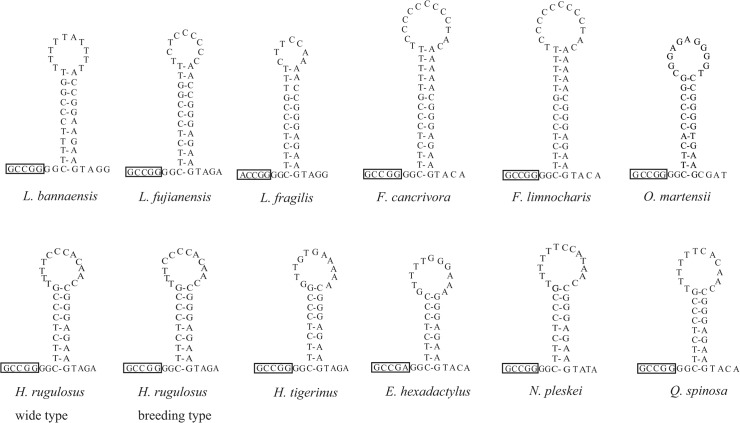
Putative secondary structures of O_L_ of 12 species of Dicroglossidae. Dashes (–) indicate Watson–Crick base pairing.

The putative origin of the L-strand replication (O_L_) [51] was located in the WANCY tRNA gene cluster between the tRNA^Asn^ and tRNA^Cys^ genes (Fig 1). This region was 30 bp long and had the potential to fold into a stem-loop secondary structure with a stem formed by 9 paired nucleotides and a loop of 12 nucleotides. In this study, we analyzed 12 sequences of O_L_ in Dicroglossidae, and found that the sequence of *H*. *rugulosus (BT)* was similar to those of *E*. *hexadactylus*, *H*. *tigerinus*, *H*. *rugulosus (WT)*, *Nanorana pleskei*, and *Quasipaa spinosa* (Fig 3).

### Phylogenetic analyses in *Hoplobatrachus*

**Fig 4:**
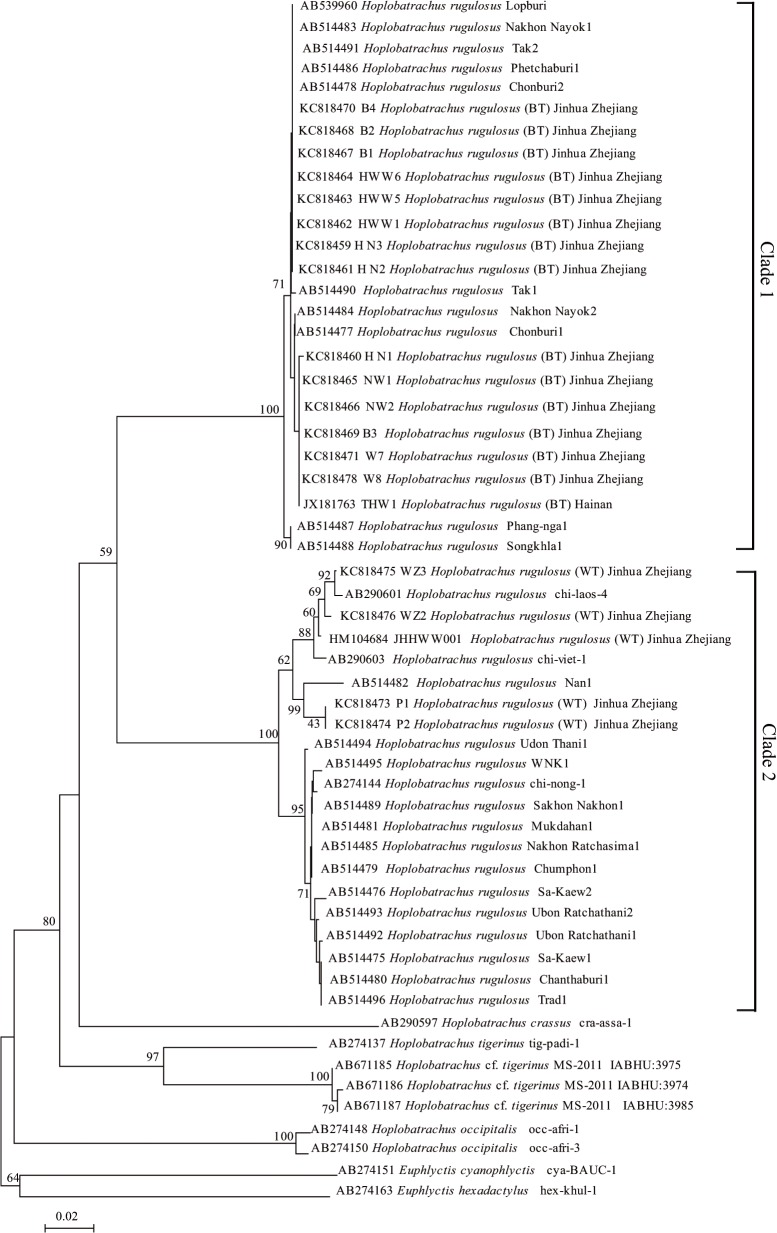
Phylogenetic relationships among *Hoplobatrachus*. Phylogenetic analysis was carried out for the 41 tiger frogs using the *Cyt b* gene. The tree was rooted with two out-groups (*E*. *cyanophlyctis* and *E*. *hexadactylus*). Numbers at the nodes are NJ bootstrap values.

Fig 4 shows the NJ phylogenetic analysis among *Hoplobatrachus* based on *Cyt b* gene. *H*. *rugulosus* was divided into two distinct clades. Chinese tiger frog (*H*. *rugulosus* WT) was clustered with the clade of *H*. *rugulosus* from northern, northeastern, and eastern Thailand. Thailand tiger frog (*H*. *rugulosus* BT) was clustered with the clade of *H*. *rugulosus* from western, central, eastern, and southern Thailand. The divergence between two clades of *H*. *rugulosus* was 11.5% using the uncorrected *p*-distance model.

**Fig 5:**
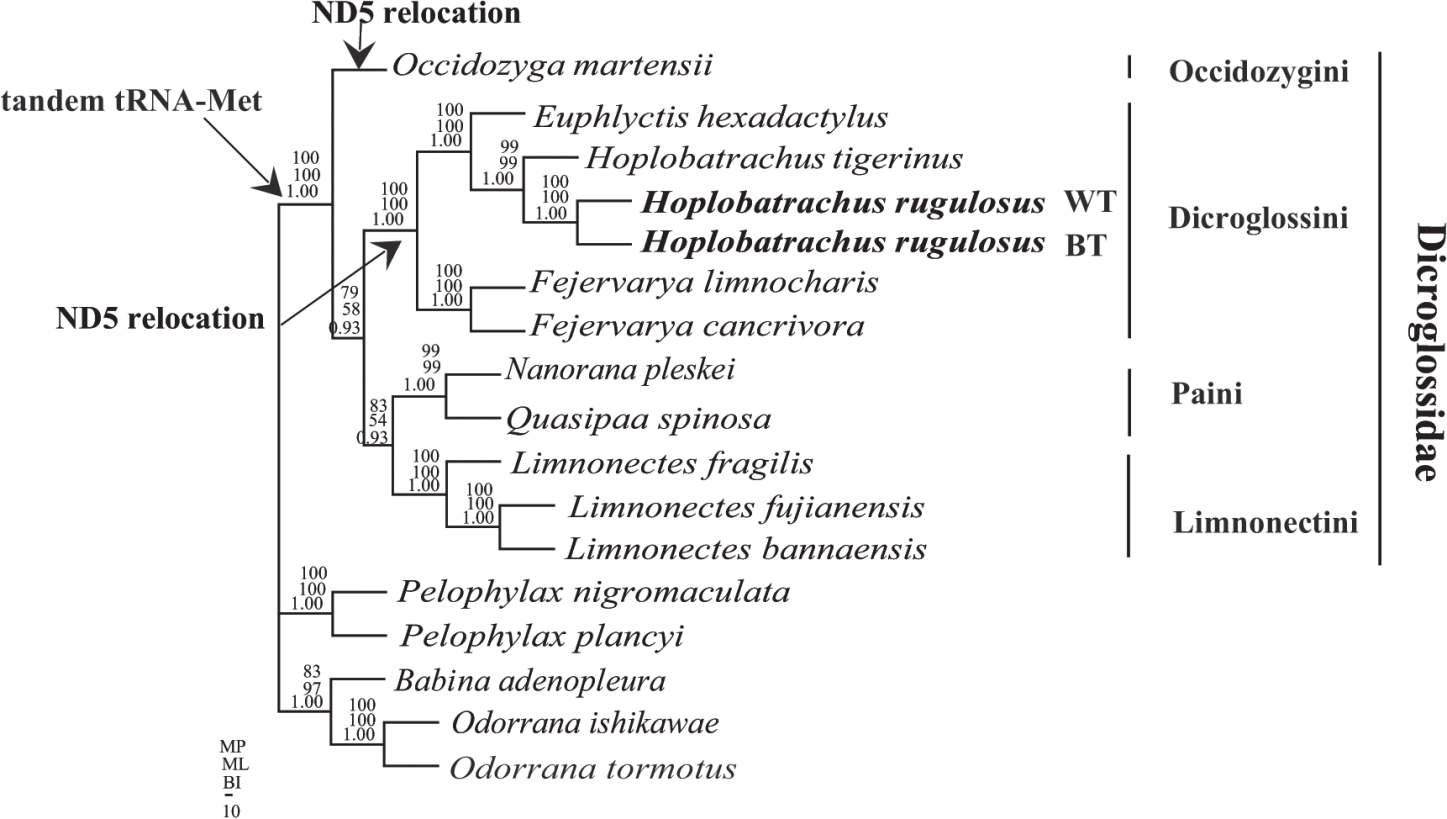
Phylogenetic relationships among Dicroglossidae and Ranidae. Phylogenetic analyses were carried out for the 17 frogs based on all 11 protein-coding genes from their respective mt genomes. The tree was rooted with five out-groups (*P*. *nigromaculata*, *P*. *plancyi*, *B*. *adenopleura*, *O*. *ishikawae*, and *O*. *tormotus*). Numbers above the nodes are the bootstrap values of MP and ML, and the posterior probabilities of BI.

BI, ML, and MP phylogenetic analyses based on the nucleotide dataset of 11 protein-coding genes had a similar topology (Fig 5), which is consistent with Zhang et al. [52]. In this study, we recovered topological relationships among dicroglossid clades with high bootstrap and posterior probability (Fig 5). *O*. *martensii* (Occidozyginae: Occidozygini) occupied the basal phylogenetic position among the dicroglossid frogs (posterior probabilities 1.00 in BI, bootstrap value 100% in ML and MP). The nucleotide dataset also favored a topology that placed *Hoplobatrachus* as a sister clade to *Euphlyctis* (posterior probabilities 1.00 in BI, bootstrap value 100% in ML and MP), and *H*. *rugulosus (BT) was a sister clade to H*. *rugulosus (WT)* (posterior probabilities 0.93 in BI, bootstrap value 54% in ML and 83% in MP).

#### Analyses in *Hoplobatrachus* using the nuclear genes

A total data of 19 sequences of the *NCX* gene with 7 variable sites of 892 nucleotides, 21 sequences of *Rag1* gene including *H*. *rugulosus* (HM163612) and *H*. *occipitalis* (HM163613) from GenBank with 20 variable sites of 783 nucleotides, 21 sequences of *Rhod* gene including *H*. *rugulosus* (AJ564731) and *H*. *tigerinus* (AB489039) from GenBank with 4 variable sites of 313 nucleotides, and 21 sequences of *Tyr* gene including *H*. *tigerinus* (AB277358) and *H*. *occipitalis* (AJ564729) from GenBank with 14 variable sites of 532 nucleotides, were used to evaluate the divergence between the Chinese tiger frog and the Thailand tiger frog, respectively. S3–S6 Tables show the nucleotide genetic divergences among the samples with *NCX1*, *Rag1*, *Rhod*, and *Tyr* genes, respectively. The nucleotide mean genetic divergences between *H*. *rugulosus* (BT) and *H*. *rugulosus* (WT) using *NCX1*, *Rag1*, *Rhod*, and *Tyr* genes were 0.7%, 0.3%, 0.1%, 0.1%, respectively. We found fixed different nucleotide sites in the *Rag1* gene: C and G in *H*. *rugulosus* (BT) but T and A in *H*. *rugulosus* (WT) on 386th and 695th site, respectively. Using NJ analysis based on the data of *Rag1*, *Rhod*, and *Tyr* genes, we also found two clades (clade 1 and 2) in *Hoplobatrachus* (S1 Fig).

### Structure analyses on ND5 gene in *Hoplobatrachus*

**Fig 6:**
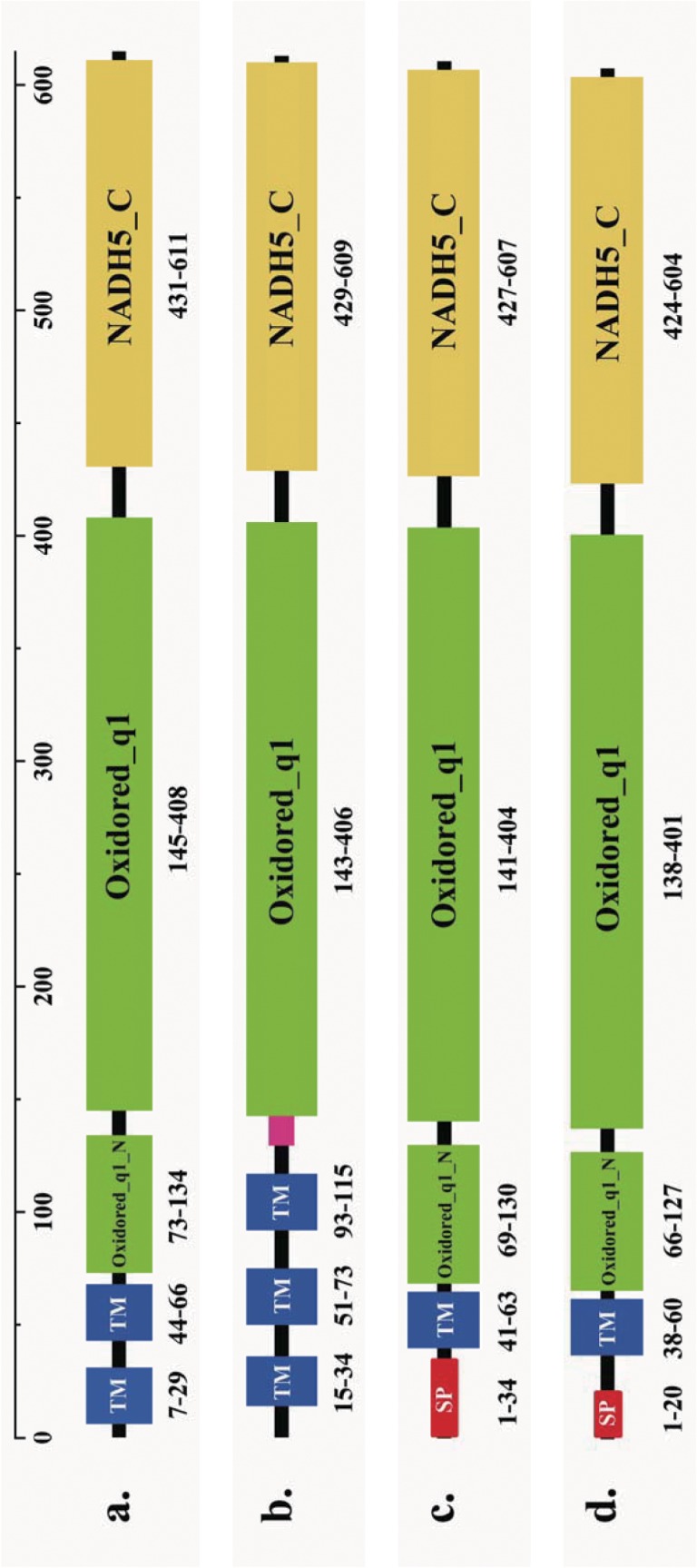
Graphic depiction of ND5 domain structure. a: Domain structure of ND5-1 from *H*. *rugulosus* (BT). b: Domain structure of ND5-2 from *H*. *rugulosus* (BT). c: Domain structure of ND5 from *H*. *rugulosus* (WT). d: Domain structure of ND5 from *H*. *tigerinus*. Signal peptides are shown in red, transmembrane regions in blue, and low complexity in pink. The Oxidored_q1_N and Oxidored_q1 regions are shown in green. The NADH5_C region is shown in yellow.

SMART program analysis revealed the presence in ND5-1 of *H*. *rugulosus (BT)* of two transmembrane domains (residues 7 to 29 in the amino acid sequence and residues 44 to 66), an Oxidored_q1_N domain (residues 73 to 134), an Oxidored_q1 domain (residues 146 to 408), and a NADH5_C domain (residues 431 to 611) (Fig 6A). Three transmembrane domains (residues 15 to 34 in the amino acid sequence, residues 51 to 73, and residues 93 to 115), an Oxidored_q1 domain (residues 143 to 406), and a NADH5_C domain (residues 429 to 609) existed in the ND5-2 of *H*. *rugulosus* (BT) (Fig 6B). A signal peptide (residues 1 to 34), a transmembrane domain (residues 41 to 63), an Oxidored_q1_N domain (residues 69 to 130), an Oxidored_q1 domain (residues 141 to 404), and a NADH5_C domain (residues 427 to 607) were present in ND5-1 and ND5-2 of *H*. *rugulosus* (WT) (Fig 6C). A signal peptide (residues 1 to 20), a transmembrane domain (residues 38 to 60), an Oxidored_q1_N domain (residues 66 to 127), an Oxidored_q1 domain (residues 138 to 401), and a NADH5_C domain (residues 424 to 604) were present in ND5-1 and ND5-2 of *H*. *tigerinus* (Fig 6D).

**Fig 7:**
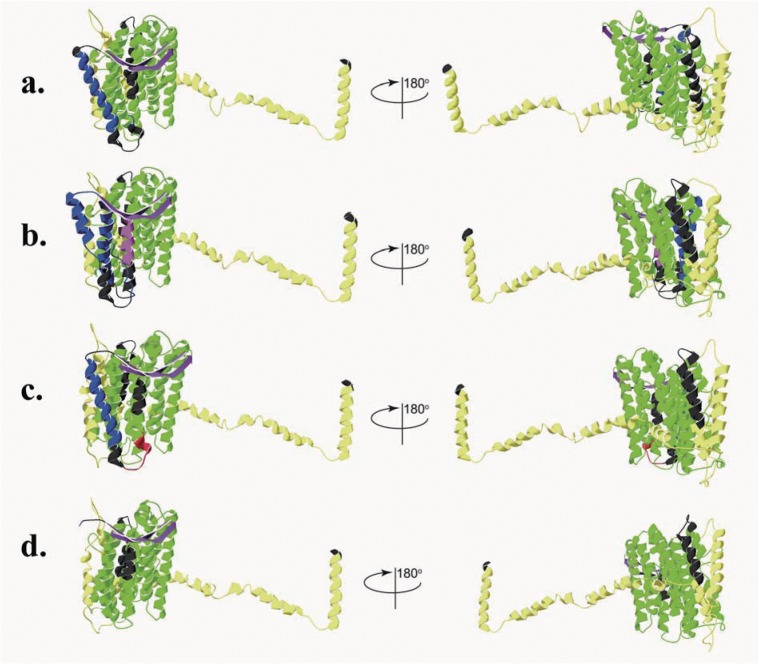
The predicted three-dimensional structures of ND5 proteins. a: Ribbon diagram structure of ND5-1 from *H*. *rugulosus* (BT). b: Ribbon diagram structure of ND5-2 from *H*. *rugulosus* (BT). c: Ribbon diagram structure of ND5 from *H*. *rugulosus* (WT). d: Ribbon diagram structure of ND5 from *H*. *tigerinus*. The signal peptide is shown in red, transmembrane regions in blue and β strands in purple. The Oxidored_q1_N and Oxidored_q1 regiona are shown in green. The NADH5_C region is shown in yellow. α helices and loops outside of the domain regions are shown in black. The low complexity region is marked in pink. Signal peptide and transmembrane regions of ND5 from *H*. *tigerinus* aer not shown in part d.

The three-dimensional structure analyses of ND5 proteins revealed that ND5-1 of *H*. *rugulosus* (BT) contained 2 β-sheet and 22 α-helices; ND5-2 of *H*. *rugulosus* (BT) contained 2 β-sheet and 23 α-helices; ND5-1 and; ND5-2 of *H*. *rugulosus (WT)* contained 2 β-sheet and 23 α-helices; and ND5-1; and ND5-2 of *H*. *tigerinus* contained 2 β-sheet and 21 α-helices (Fig 7). β-Sheet and α-helices were attached to one another by relatively flexible, highly charged loops (Fig 7). The overall model quality Z-scores were—5.82 for ND5-1 of *H*. *rugulosus* (BT); –4.77 for ND5-2 of *H*. *rugulosus* (BT); –5.61 for ND5-1 and ND5-2 of *H*. *rugulosus (WT);* and −6.27 for ND5-1 and ND5-2 of *H*. *tigerinus*. Local model quality values were generally below zero for all ND5 proteins. The 3D–1D averaged score was generally distributed from—0.55 to 0.91 for ND5-1 of *H*. *rugulosus* (BT); from—0.46 to 0.86 for ND5-2 of *H*. *rugulosus* (BT); from—0.6 to 0.92 for ND5-1 and ND5-2 of *H*. *rugulosus (WT);* and from—0.51 to 0.79 for ND5-1 and ND5-2 of *H*. *tigerinus*.

## Discussion

### Evidence of cryptic species

The divergences of partial *Cyt b* gene and mt genome in *H*. *rugulosus*. The taxonomic problem of H. rugulosus has been debated by a number of researchers [10,13,16,53]. Alam et al. [16] and Pansook et al. [17] found high sequence divergences in H. rugulosus. In our study, we found high divergence (13.8%) between H. rugulosus WT and BT using the Cyt b gene, and high divergence (11.5%) between two different clades (clade 1 and 2) of the phylogenetic relationship in H. rugulosus (Fig 4). Based on the Cyt b gene divergence we support the suggestion [17] that H. rugulosus is a cryptic species complex.

Nucleotide divergence within the complete mt genome was 14.0% between *H*. *rugulosus* WT and BT, which is very high but not beyond the genetic divergence found in cryptic species of diverse other taxa such as *Ciona intestinalis*, *Friesea grisea*, and *Taenia taeniaeformis* [7–9]. Deep nucleotide divergence in protein-coding genes (11.2–24%) was also found using the uncorrected *p*-distance model between *H*. *rugulosus* (WT) and *H*. *rugulosus* (BT), which is similar to the values for cryptic species in some frogs [18,20].

The two ND5 genes in *H*. *rugulosus* (WT) and *H*. *tigerinus* were identical within each species whereas the two ND5 genes in *H*. *rugulosus (BT)* had only 84.1% similarity sequence. Although the transmembrane domains and three-dimensional structure analyses of ND5 proteins had differences among *H*. *tigerinus*, *H*. *rugulosus (BT)*, *and H*. *rugulosus* (WT), the two ND5 genes in *H*. *rugulosus* (WT) had an identical tertiary structure (Fig 7). The predicted results (Fig 7) showed that ND5-1 and ND5-2 of *H*. *rugulosus (BT)* had a similar structural plan, which suggested a common origin in the two ND5 genes. A comparison of all four types of ND5 genes in *Hoplobatrachus*, the protein structure of the two ND5 genes in *H*. *rugulosus (BT)* differed from those in *H*. *rugulosus (WT) and H*. *tigerinus*, which suggested that *H*. *rugulosus (BT)* diverged from *H*. *rugulosus (WT)* in evolution. According to the different nucleotide divergences, the transmembrane domains, three-dimensional structure, and gene arrangement of the ND5 gene between *H*. *rugulosus* (WT) and *H*. *rugulosus (BT)*, *H*. *rugulosus (WT) and H*. *rugulosus (BT)*, *two different duplication of ND5 genes* may have been independently formed in phylogenetic evolution.

Based on the mt genome divergence of the total nucleotide and protein-coding genes (Table 4), as well as the character of ND5 gene in *H*. *rugulosus (BT)*, we can also conclude that *H*. *rugulosus is a cryptic species complex*. *Yet*, *we failed to find the high genetic divergence between H*. *rugulosus (BT) and H*. *rugulosus* (WT) using *NCX1*, *Rag1*, *Rhod*, and *Tyr* genes.

#### mt genome rearrangement in *H*. *rugulosus* (BT)

In all published dicroglossid sequences, tRNA^Leu (CUN)^, tRNA^Thr^, tRNA^Pro^, and tRNA^Phe^ genes were translocated from their original position of Archaeobatrachia and formed a LTPF tRNA gene cluster the upstream of the 12S rRNA gene. However, the members and arrangement of this tRNA gene cluster are slightly modified in some taxa [21,54]. In our study, the tRNA^Leu(CUN)^ gene was found between the duplicated D-loop-ND5 regions as previously observed in *H*. *tigerinus* [21] and H. rugulosus (WT) [22]. A TPF tRNA gene cluster at the upstream of 12S rRNA gene was formed, which was consistent with *H*. *tigerinus* [21] and *H*. *rugulosus* (WT) [22]. We analyzed the four samples of Thailand tiger frogs by amplifying sequences from ND5 to 12S RNA genes, and the TPF tRNA gene cluster was also found. Thus, the TPF tRNA gene cluster can be regarded as a synapomorphy of *Hoplobatrachus*, which was consistent with the finding of Alam et al. [21]. *Zhou et al*. [5], Alam et al. [21], and Chen et al. [55] suggested that the tandem duplication of tRNA^Met^ gene can be regarded as a synapomorphy of Dicroglossinae. In our results, tandem tRNA^Met^ genes were found to exist in *H*. *rugulosus (BT)*.

Identical tandem duplication of ND5-D-loop was observed in *H*. *tigerinus and H*. *rugulosus (WT)*, *but only a similar tandem duplication of* ND5-D-loop was observed in *H*. *rugulosus (BT)*. The possible rearrangement pathway of the two different ND5 genes in *H*. *rugulosus (BT)* can be explained by the random duplication model as well as *H*. *tigerinus* [21]. Yet, the random loss has not happened in two ND5-D-loop regions. The mutation in two ND5 genes of *H*. *rugulosus (BT)* maybe happened dependently by some unknown selection pressures during the evolution history. The different arrangement pathways of the ND5 gene and the three-dimensional structure of ND5 proteins in *H*. *rugulosus (BT) also* suggested that *H*. *rugulosus* represents a cryptic species complex comprising at least two well supported lineages (BT and WT).

### mt genome rearrangement and phylogeny of Dicroglossidae

The phylogenetic relationships among dicroglossids were mostly similar to the previous molecular phylogeny [5,52,56]. Although the phylogenetic topology is the similar to Zhang et al [52], the bootstrap values of MP, ML and NJ and the posterior probabilities of BI are well supported (Fig 5)In the complete mtDNA of dicroglossids retrieved from GenBank, ND5 gene translocation was observed in *O*. *martensii*, *Fejervarya limnocharis*, *Fejervarya cancrivora*, *E*. *hexadactylus*, *H*. *rugulosus* (WT), and *H*. *tigerinus*. In this study, the location of two ND5 genes of *H*. *rugulosus (BT)* was consistent with *H*. *tigerinus* and *H*. *rugulosus* (WT) [21,22]. We identified all different mt genomes of anurans in GenBank. ND5 gene translocation occurred in three distinct lineages of focusing on families closely related to Dicroglossidae: the lineage to *O*. *martensii* (Occidozyginae), the lineages to Mantellidae and Rhacophoridae, as well as the lineages to *Fejervarya*, *Euphlyctis*, and *Hoplobatrachus* (Dicroglossinae).

Generally, mt genome arrangements are believed to reflect phylogenetic relationships [57–59]. Using such information, three major lineages can be separated in Dicroglossidae: translocation of ND5, LTPF tRNA gene cluster, and tandem duplication of tRNA^Met^, which were observed in *O*. *martensii* (Occidozyginae). The LTPF tRNA gene cluster and the tandem duplication of tRNA^Met^ were observed in *Quasipaa*, *Nanorana*, and *Limnonectes*. The translocation of ND5, the modified LTPF tRNA gene cluster, and the tandem duplication of tRNA^Met^ were observed in *Fejervarya*, *Hoplobatrachus*, and *Euphlyctis*. The evolutionary relationships of dicroglossid taxa indicated in the phylogenetic trees, which was based on the concatenated sequences of the 11 protein coding genes (Fig 4), were similar to the traditional classification [14,54,60–63]. Based on the results of genome rearrangement and phylogenetic relationship in dicroglossids, we found that the genome rearrangement of Dicroglossidae was consistent with the results of phylogenetic analyses (Fig 4).

Although NJ analysis based on the data of *Rag1*, *Rhod*, and *Tyr* genes, two clades (clade 1 and 2) in *Hoplobatrachus* (S1 Fig) were supported, the genetic divergences of those genes is low. Our findings highlight the need for further nuclear gene and crossbreeding studies of *H*. *rugulosus* (WT) and *H*. *rugulosus* (BT). Whether cryptic species exist or not in *H*. *rugulosus*, Thailand tiger frogs (*H*. *rugulosus* (BT)) should be prevented from escaping into wild environments to protect Chinese tiger frogs. We strongly advocate that Thailand tiger frogs in the farms should be strictly managed and that release of these frogs into the wild should be prohibited lest they produce hybrids with Chinese tiger frogs.

## Conclusion

Based on the *Cyt b* gene divergence (13.8%) between *H*. *rugulosus* WT and BT, the mt genome divergence of the total nucleotide (14.0%) between *H*. *rugulosus* WT and BT, different three-dimensional structure of ND5 proteins and different rearrangement pathways of the ND5 gene between *H*. *rugulosus* WT and BT, all suggest that *H*. *rugulosus* is a cryptic species complex. Although using the nuclear gene (*NCX1*, *Rag1*, *Rhod*, and *Tyr* genes), we failed to find the high genetic divergence between *H*. *rugulosus* WT and BT, we found two clades (clade 1 and 2) in *Hoplobatrachus* (S1 Fig) using NJ analysis based on the data of *Rag1*, *Rhod*, and *Tyr* genes. Because of the genetic difference between *H*. *rugulosus* WT and BT, we suggest that we should prevent Thailand tiger frogs from escaping into wild environments lest they produce hybrids with Chinese tiger frogs.

## Supporting Information

S1 Fig Phylogenetic relationships among *Hoplobatrachus* using nuclear genes. Phylogenetic analysis was carried out for the 20 tiger frogs using *Rag 1*, *Rhod* and *Tyr* gene. The tree was rooted with a chimeric sequence combining data from two species (*H*. *tigerinus* with *Rhod* and *Tyr*, *H*. *occipitalis* with *Rag 1*). Numbers at the nodes are NJ bootstrap values.
(EPS)

S1 Table Information about the *Cyt b* gene in species with GenBank accession numbers used in this study. (DOC)

S2 Table List of species used in this study, along with GenBank accession numbers and A+T content of total mitochondrial genome and control region (D-loop). (DOC)

S3 Table List of the nucleotide genetic divergences among 19 samples of *NCX* gene in species used in this study. The meaning of symbols is shown in S2 Table.
(XLS)

S4 Table List of the nucleotide genetic divergences among 21 samples of *Rag 1* gene in species used in this study including *H*. *rugulosus* (HM163612) and *H*. *occipitalis* (HM163613). The meaning of symbols is shown in S2 Table.
(XLS)

S5 Table List of the nucleotide genetic divergences among 21 samples of *Rhod* gene in species used in this study including *H*. *rugulosus* (AJ564731) and *H*. *tigerinus* (AB489039). The meaning of symbols is shown in S2 Table.
(XLS)

S6 Table List of the nucleotide genetic divergences among 21 samples of *Tyr* gene in species used in this study including *H*. *tigerinus* (AB277358) and *H*. *occipitalis* (AJ564729). The meaning of symbols is shown in S2 Table.
(XLS)
