# Supplementary material for: Do Cryptic Species Exist in Hoplobatrachus rugulosus? An Examination Using Four Nuclear Genes, the Cyt b Gene and the Complete MT Genome
Source: PLoS One. 2015 Apr 13;10(4):e0124825. doi: 10.1371/journal.pone.0124825 (PMC4395372; doi:10.1371/journal.pone.0124825)
Supplement: S1 Table — (DOC) [file pone.0124825.s002.doc]

**Table S1 List of the information of *Cyt* *b* gene in species with GenBank accession numbers used in this study.**

| Species (No. of Samples) | Accession No. | locations of samples | References |
| --- | --- | --- | --- |
| *E. cyanophlyctis* | AB274151 |  | Alam et al 2008 |
| *E. hexadactylus* | AB274163 |  | Alam et al 2008 |
| *H. occipitalis* | AB274148, AB274150 |  | Alam et al 2008 |
| *H. crassus* | AB290597 |  | Alam et al 2008 |
| *H. rugulosus* | AB274144, AB290601, AB290603 |  | Alam et al 2008 |
| *H. rugulosus* | AB514475-AB514496, AB539960 |  | Pansook et al 2012 |
| *H. tigerinus* | AB274137 |  | Alam et al 2008 |
| *H.* cf. *tigerinus* | AB671185-AB671187 |  | Hasan et al 2012 |
| *H. rugulosus* (JHHWW001) | HM104684 | wild field in Jinhua | Yu et al. 2012 |
| *H. rugulosus* (THW1) | JX181763 | the Huwawa farm in Jinhua, | This study |
| *H. rugulosus* (HNBT-HN3) | KC818459 | the Hainan farm in Jinhua | This study |
| *H. rugulosus* (HNBT-HN2) | KC818460 | the Hainan farm in Jinhua | This study |
| *H. rugulosus* (HNBT-HN1) | KC818461 | the Hainan farm in Jinhua | This study |
| *H. rugulosus* (JHBT-HWW1) | KC818462 | the Huwawa farm in Jinhua, | This study |
| *H.rugulosus* (JHBT-HWW5) | KC818463 | the Huwawa farm in Jinhua, | This study |
| *H. rugulosus* (JHBT-HWW6) | KC818464 | the Huwawa farm in Jinhua, | This study |
| *H. rugulosus* (JHBT-NW1) | KC818465 | the Niuwa farm in Jinhua | This study |
| *H. rugulosus* (JHBT-NW2) | KC818466 | the Niuwa farm in Jinhua | This study |
| *H. rugulosus* (JHBT-B1) | KC818467 | the Bendi farm in Jinhua | This study |
| *H. rugulosus* (JHBT-B2) | KC818468 | the Bendi farm in Jinhua | This study |
| *H. rugulosus* (JHBT-B3) | KC818469 | the Bendi farm in Jinhua | This study |
| *H. rugulosus* (JHBT-B4) | KC818470 | the Bendi farm in Jinhua | This study |
| *H. rugulosus* (JHBT-W7) | KC818471 | the Wu farm in Jinhua | This study |
| *H. rugulosus* (JHBT-W8) | KC818472 | the Wu farm in Jinhua | This study |
| *H. rugulosus* (PAWT-P1) | KC818473 | wild field in Panan country | This study |
| *H. rugulosus* (PAWT-P2) | KC818474 | wild field in Panan country | This study |
| *H. rugulosus* (JHWT-WZ3) | KC818475 | wild field in Jinhua | This study |
| *H. rugulosus* (JHWT-WZ2) | KC818476 | wild field in Jinhua | This study |
