# Supplementary material for: Do Cryptic Species Exist in Hoplobatrachus rugulosus? An Examination Using Four Nuclear Genes, the Cyt b Gene and the Complete MT Genome
Source: PLoS One. 2015 Apr 13;10(4):e0124825. doi: 10.1371/journal.pone.0124825 (PMC4395372; doi:10.1371/journal.pone.0124825)
Supplement: S2 Table — (DOC) [file pone.0124825.s003.doc]

**Table S2** List of species used in this study, along with GenBank accession numbers and A+T content of total mitochondrial genome and control region (D-loop).

| Species | Accession | A+T | |
| --- | --- | --- | --- |
|  | number | Total | D-loop |
| Ranidae |  |  |  |
| *Babina adenopleura* | JX033120 | 58.9 | 70.2 |
| *Pelophylax nigromaculata* | AB043889 | 56.9 | 65.4 |
| *Pelophylax plancyi* | EF196679 | 56.6 | 63.4 |
| *Odorrana tormotus* | DQ835616 | 57.9 | 64.3 |
| *Odorrana ishikawae* | AB511282 | 59.3 | 70.1 |
| Dicroglossidae |  |  |  |
| *Euphlyctis hexadactylus* | [AP011544](http://www.ncbi.nlm.nih.gov/nuccore/AP011544) | 57 | 57.1/58 |
| *Fejervarya cancrivora* | EU652694 | 56.7 | 59.2 |
| *Fejervarya limnocharis* | AY158705 | 58.0 | 63.4 |
| *Hoplobatrachus rugulosus* (WT) | HM104684 | 53.1 | 54.2 |
| *Hoplobatrachus rugulosus* (BT) | JX181763 | 52.8 | 56.6/57.0 |
| *Hoplobatrachus tigerinus* | [AP011543](http://www.ncbi.nlm.nih.gov/nuccore/AP011543) | 55.4 | 54.9/55.8 |
| *Limnonectes fujianensis* | AY974191 | 57.6 | 62.3 |
| *Limnonectes bannaensis* | AY899242 | 58.6 | 60.1 |
| *Limnonectes fragilis* | NC_016066 | 57.9 | 61.6 |
| *Nanorana pleskei* | NC_016119 | 58.9 | 58.3 |
| *Occidozyga martensii* | [GU177877](http://www.ncbi.nlm.nih.gov/nuccore/GU177877) | 62.3 | 65.9 |
| *Quasipaa spinosa* | FJ432700 | 59.6 | 66.8 |
